# Measuring repeatability of compositional diet estimates: An example using quantitative fatty acid signature analysis

**DOI:** 10.1002/ece3.9428

**Published:** 2022-10-27

**Authors:** Connie Stewart, Shelley L. C. Lang, Sara Iverson, W. Don Bowen

**Affiliations:** ^1^ Department of Mathematics and Statistics University of New Brunswick Saint John Saint John New Brunswick Canada; ^2^ Department of Biology Dalhousie University Halifax Nova Scotia Canada; ^3^ Fisheries and Oceans Canada Bedford Institute of Oceanography Dartmouth Nova Scotia Canada; ^4^ Present address: Northwest Fisheries Sciences Centre Fisheries and Oceans Canada St. John's Newfoundland and Labrador Canada

**Keywords:** bootstrap confidence intervals, Chi‐square distance, grey seal, *Halichoerus grypus*, intraclass correlation coefficient, nonparametric, predator diet, quantitative fatty acid signature analysis, repeatability

## Abstract

By measuring the temporal consistency, or repeatability, in the diets of predators, we can gain a better understanding of the degree of individual specialization in resource utilization and implications for predator–prey interactions, population dynamics, and food web structure. To measure repeatability, we require repeated diet estimates of individuals over time, such as those derived from quantitative fatty acid signature analysis (QFASA), a popular diet estimation technique. However, diet estimates are often lengthy compositional vectors with many zeros, as some prey will not be consumed by all individuals, precluding the use of previously proposed measures of repeatability. In this paper, we propose a novel approach for inferring repeatability for multivariate data and, in particular, compositional diet estimates. We extend the commonly used measure of repeatability for univariate data to the multivariate compositional setting by utilizing the mean squares obtained from a nonparametric multivariate analysis of variance, and an appropriate choice of statistical distance. Our measure and its extension are compatible with both balanced and unbalanced data sets. Associated confidence intervals via nonparametric bootstrapping are also developed for the case of QFASA diet estimates that incorporate both sampling error and measurement error, where the latter error arises because the diets of predators are estimated. Simulation study results suggest that for practical levels of repeatability, our methods yield confidence intervals with the desired coverage probability even when the sample size relative to the dimension of the data (i.e., number of prey species eaten) is small. We tested our methods using QFASA diet estimates for free‐ranging Northwest Atlantic grey seals. Given the importance of understanding how predator diets vary over time and space, our method may find broad application to other compositional diet estimates, including those derived from the stomach or fecal contents, and stable isotope analyses.

## Graphical abstract

By measuring the temporal consistency, or repeatability, in the diets of predators, we can gain a better understanding of the degree of individual specialization in resource utilization and implications for predator‐prey interactions, population dynamics, and food web structure. We present a novel way of inferring repeatability for multivariate data and, in particular, compositional diet estimates. Our methods can accommodate problematic zeros in the data, small sample sizes, and missing data. Given the importance of understanding how predator diets vary over time and space, our method may find broad application.

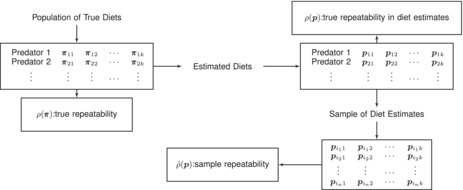

## 1 INTRODUCTION

Estimates of predator diets are central to understanding many areas of ecology, such as predation and the structure of food webs. Although diets of animal populations are frequently presented as averages with individuals of a given age, sex, or morphology treated as ecologically equivalent, individuals within a population can vary substantially in their resource use (Araújo et al., 2011; Bolnick et al., 2003, 2011; Estes et al., 2003). Such individual specialization, whereby individuals use only a subset of the population's resource base, is of considerable interest because of its potential to profoundly affect the structure and dynamics of populations and their communities (Araújo et al., 2011; Bolnick et al., 2011).

One of the challenges associated with understanding the empirical importance of individual specialization, and the factors that may influence it, is identifying the time scale over which such specialization occurs (Layman et al., 2015; Novak & Tinker, 2015). For species with large home ranges or foraging areas that are not readily observable, obtaining sufficient numbers of repeated observations of diet compositions to characterize levels of temporal consistency within individuals may be difficult or impossible. For these species, methods that integrate dietary information over longer periods (weeks to months), such as isotopic signatures or fatty acid (FA) profiles, can be used to overcome this limitation (Araújo et al., 2011; Bolnick et al., 2003).

Quantitative fatty acid signature analysis (QFASA, Iverson et al., 2004) is now a widely applied approach to estimating a predator's diet by comparing the FA profiles of metabolically active fat stores of predators with that of their potential prey, after taking into account modifications due to FA metabolism in the predator. For tissues such as blubber or adipose, which contain FAs that have accumulated over time, QFASA can provide an integrated record of dietary intake over a period of weeks to months (Budge et al., 2006) and has been used to estimate diets for a wide range of marine species (Zhang et al., 2020) including fish (Magnone et al., 2015), seabirds (Haynes et al., 2015; Iverson et al., 2007), pinnipeds (Beck et al., 2007; Bromaghin et al., 2013; Meynier et al., 2010), and polar bears (Galicia et al., 2016; Iverson et al., 2006; Thiemann et al., 2008). In cases where individual predators can be repeatedly sampled, diets estimated using QFASA provide an opportunity to examine temporal consistency over multiple time scales (e.g., Thiemann et al., 2011).

However, assessing the temporal consistency of QFASA diet estimates is complicated by the structure of the estimate itself. QFASA yields an estimate of the proportion of each prey species in the predator's diet. The sum constraint of the QFASA estimate (the values must sum to 1) restricts the application of common indices of diet similarity, such as the proportion similarity index (see Novak & Tinker, 2015; Powell & Taylor, 2017), since the resampling procedures used for hypothesis testing cannot be used on estimates that are purely compositional. Although measures such as the chi‐squared contingency analysis (Estes et al., 2003 or Thiemann et al., 2011) dietary change index have been used to examine individual consistency in concurrent, compositional diet estimates over different time scales, these analysis methods are limited to the comparison of within‐individual variation only and, therefore, do not incorporate the variance in resource use associated with the population. Here, we propose a statistical approach for assessing the temporal consistency in QFASA diet estimates using an extension of univariate repeatability (that is, repeatability computed for data collected on a single variable), which accounts for the compositional nature of the estimates and the presence of essential zeros (zeros corresponding to the absence of a particular prey item in the diet of an individual). Repeatability is defined as the proportion of total variation in measurements that can be ascribed to variation among individuals rather than the variation among measurements within individuals (Wolak et al., 2012). Consequently, repeatability can simultaneously incorporate the variance in resource use associated with the individual and the population and provide insight into the extent to which measurements are characteristic of individuals. Higher repeatability estimates can indicate that there is more variation among individuals than within individuals (Lessells & Boag, 1987), suggesting that there is temporal consistency in resource use within individuals.

While various approaches exist to measure repeatability (see Wolak et al., 2012 for a list of references), it is commonly estimated by the intraclass correlation coefficient (ICC). In the case of measurements on a single variable (or univariate measurements), where the only systematic source of variability occurs among individuals, Lessells and Boag (1987) provide the widely accepted formula for estimating ICC. When the observations within individuals differ in some systematic way, such as through a possible season or year effect (see McGraw & Wong, 1996 for a more extensive discussion on what constitutes a systematic source of variance), a two‐way model (a model with both row and column effects or, equivalently, a model with two factors) is more appropriate. Estimating ICC for the univariate two‐way case is discussed in McGraw and Wong (1996), and our approach is an extension of this work. We propose obtaining a point estimate of repeatability using the mean squares computed from the nonparametric multivariate analysis of variance (MANOVA) developed by Anderson (2001). The nonparametric MANOVA requires the calculation of distances between the multivariate responses. To handle the compositional diet vectors and the essential zeros, we use the chi‐square (CS) measure of distance, as recommended in Stewart (2017) for QFASA applications. While the extension of the ICC definitions in McGraw and Wong (1996), reviewed in Section 2.1, to the multivariate setting in this manner is relatively straightforward, we are not aware of repeatability being computed in this way previously. We consider the balanced case in which there are no missing values and the number of measurements (diet estimates) per individual is constant, as well as the unbalanced case in which the number of measurements per individual is allowed to vary.

In addition to the point estimation of repeatability, we consider the development of confidence intervals (CIs) that properly reflect the various sources of variability in the QFASA diet estimates. Although rarely estimated, exact CIs for the population value of ICC based on the *F* distribution have been available for some time (Wolak et al., 2012). For non‐normal univariate data with individuals or, more generally, clusters as the only factor (analogous to the one‐way ANOVA setting), Ukoumunne et al. (2003) proposed using nonparametric bootstrap CIs involving a variance stabilizing transformation. In our work, nonparametric bootstrapping is also used to provide CIs for the true repeatability in a population.

Using simulated datasets, we examine the performance of our proposed measure of repeatability and associated confidence intervals (CIs) with respect to coverage probability and confidence interval lengths (where the length of the intervals reflects how precisely we can estimate repeatability) and then apply our methods for both balanced and unbalanced designs to QFASA diet estimates from free‐ranging northwest Atlantic grey seals (*Halichoerus grypus*). A further statistical complexity in the unbalanced case is that the sample size is small relative to the dimension of the diet estimates. The grey seal is an upper‐trophic level marine predator that inhabits temperate waters on both sides of the North Atlantic Ocean. In the Northwest Atlantic, the grey seal has a broad continental shelf distribution from the Gulf of Maine north to the Gulf of St. Lawrence with the largest breeding colony on Sable Island (den Heyer et al., 2021). Adult grey seals on Sable Island make repeated foraging trips to shallow offshore banks on the Eastern Scotian Shelf with a few traveling into the Gulf of St. Lawrence and south to the Gulf of Maine (Austin et al., 2006; Breed et al., 2006, 2009; Lidgard et al., 2012). A fine‐scale spatial and temporal analysis of the movements of adults provided clear evidence of within‐year fidelity to presumed foraging locations, suggesting some levels of predictability in prey distribution and possibly diet (Lidgard et al., 2020). Repeated tracking of 21 adults also indicates that individuals exhibit similar movements and foraging distributions over years further suggesting that there may be temporal consistency in the diet (W. D. Bowen and C. E. den Heyer, unpublished data). While this work focuses on calculating repeatability and its CIs for diets estimated by QFASA, it can be extended to other multivariate data sets, including diet estimates derived from other methods, provided an appropriate distance measure is chosen.

## 2 METHODS

### 2.1 Point estimation of repeatability

To set the notation, consider the univariate balanced setting in which we have, in concept, a population of predators and for each, *k* measurements taken over time. Due to only having one observation per treatment (or “cell”), we make the necessary assumptions that there is no interaction between the predators and the time points, and that the levels of the time factor are fixed. Note, however, that this latter assumption has no effect on the definition of ICC or its estimator, but the interpretation and generalization may depend on whether the levels are actually fixed or random. Following McGraw and Wong (1996), we define the ICC (denoted by *ρ*) for this two‐way model setting as

(1) $$\rho =\frac{\sigma_{s}^{2}}{\sigma_{s}^{2}+\theta_{t}+\sigma_{e}^{2}},$$

where $\sigma_{s}^{2}$ denotes the variability in the univariate seal measurements, $\theta_{t}=\sqrt{t_{j}^{2}/\left(k-1\right)}$ with *t*_*j*_ denoting the *j*th time effect, and $\sigma_{e}^{2}$ is the variability in the residual effects.

For a random sample of *n* predators with *k* measurements per predator, the estimate of *ρ* is based on the mean squares chosen in such a way that substituting their expectation (that is, replacing them with their population average) yields *ρ*. For the two‐way mixed effect model, McGraw and Wong (1996) provide the following estimate of *ρ*:

(2) $$\hat{\rho }=\frac{{\mathrm{MS}}_{s}-{\mathrm{MS}}_{e}}{{\mathrm{MS}}_{s}+\left(k-1\right){\mathrm{MS}}_{e}+\frac{k}{n}\left({\mathrm{MS}}_{t}-{\mathrm{MS}}_{e}\right)}$$

where the mean squares can be obtained from the output of a traditional randomized block two‐way ANOVA.

For the analogous multivariate setting where measurements are now *M* dimensional vectors, an estimate of *ρ* can be deduced by defining the sums of squares as distances between the pertinent multivariate predator measurements. This approach for computing sums of squares is the basis of the nonparametric MANOVA proposed in Anderson (2001), a widely accepted method of carrying out a MANOVA in the ecological community, particularly when the data do not meet the traditional MANOVA assumption requirements such as multivariate normality. An advantage of the nonparametric MANOVA is that the computed pseudo *F* statistic relies only on a symmetric distance (or dissimilarity) matrix, and any distance (or dissimilarity) measure can be used. While not needed for the calculation of *ρ*, permutations are used to determine the distribution of the *F* statistic and to test whether factors are significant. The function *adonis* in the R package *vegan* (Oksanen et al., 2017) performs the permutational MANOVA, as it is often called. For a random sample of *n* predators, each with *k* multivariate repeated measurements, we then estimate *ρ* using Equation (2) with the mean square values derived from the *adonis* output, which, in turn, requires the computation of a distance matrix between all predator measurements.

In our example data sets, the predator measurements for the *i*th individual are QFASA diet estimates, henceforth denoted as $p_{i1},\ldots ,p_{\mathrm{ik}}$, where the *m*th component of $p_{\mathrm{ij}}$ (denoted $p_{\mathrm{ijm}}$) is the QFASA estimate of $\pi_{\mathrm{ijm}}$, the true proportion of species *m* in the diet of the individual *i* at time *j*. Note that i=1,…,n and j=1,…,k. Details of the QFASA model can be found in Iverson et al. (2004). Briefly, QFASA uses a library of FA profiles (referred to as “signatures”), which are vectors of proportions that summarize the FA composition of individual predator and prey lipids. Calibration coefficients, derived from controlled feeding studies, are used to account for the differential metabolism of ingested FAs by predators. Following the application of the calibration coefficients, the model estimates the mixture of mean prey FA signatures that minimizes a statistical measure of distance between the modeled and observed predator signature. This proportional mixture is then weighted by the proximate fat content (i.e., relative FA contribution) of each prey species to estimate their proportions in the predator's diet.

In order to compute $\hat{\rho }$ for QFASA diet estimates using the nonparametric MANOVA methodology, we require a measure of distance suitable for compositional data. While, in general, the recommended distance measure for compositional data is Aitchison's distance (Martín‐Fernández et al., 1998), this distance measure involves logarithms and hence is not compatible with compositional data such as ours where there is an abundance of zeros, each arising from an estimated absence of a species in the predator's diet. Recently Stewart (2017) proposed using the CS distance to measure the distance between compositional data with zeros, and in particular QFASA diet estimates, and it is, consequently, the measure of distance that we have chosen to adopt for this application. The CS distance between two diet estimates, say ***p***_1_ and ***p***_2_ were defined in Stewart (2017) as

(3) $$\mathrm{CS}\left(p_{1},p_{2}\right)=\sqrt{2M}{\left(\sum_{m=1}^{M}r_{m}\right)}^{1/2},$$

where

$$r_{m}=\left\{\begin{array}{ll} 0 & \text{if}\;p_{1m}=p_{2m}=0\\ \frac{{\left(\frac{p_{1m}}{\sum_{c=1}^{M}p_{1c}}-\frac{p_{2m}}{\sum_{c=1}^{M}p_{2c}}\right)}^{2}}{\frac{p_{1m}}{\sum_{c=1}^{M}p_{1c}}+\frac{p_{2m}}{\sum_{c=1}^{M}p_{2c}}} & \text{otherwise}. \end{array}\right.$$

Calculation of the CS distance in R can be carried out using the function *chisq.dist* in the package *QFASA* (Stewart et al., 2021). Note the CS distance in Equation (3) does not involve a “column standardization” over predator measurements and so is different from the CS distance used in correspondence analysis (Greenacre, 2011) and cited in some ecological publications (see Warton et al., 2012, for example).

As yet we have only discussed the balanced case in which *k* is fixed for each predator. When missing values occur (as is the case for the second data set in Section 3.1), we propose using an average *k* value, similar to the approach used by Lessells and Boag (1987) for the simple univariate one‐way ANOVA setting. Lessells and Boag (1987) do not recommend the arithmetic mean but rather a modified value that reduces to *k* in the balanced setting. Sokal and Rohlf (2012) have more recently proposed using the harmonic mean in this case (see Chapter 9, p. 212), and we have chosen to use this representative value of *k* here. In addition to adjusting the value of *k* in Equation (2), we also need to modify the denominator, specifically the degrees of freedom, in the mean square formulae since they rely on *k*. We propose the following estimator of *ρ* in the unbalanced setting, which incorporates these changes:

(4) $$\tilde{\rho }=\frac{{\mathrm{SS}}_{s}/{\mathrm{df}}_{s}-{\mathrm{SS}}_{e}/{\mathrm{df}}_{e}}{{\mathrm{SS}}_{s}/{\mathrm{df}}_{s}+\left(\tilde{k}-1\right){\mathrm{SS}}_{e}/{\mathrm{df}}_{e}+\frac{\tilde{k}}{n}\left({\mathrm{SS}}_{t}/{\mathrm{df}}_{t}-{\mathrm{SS}}_{e}/{\mathrm{df}}_{e}\right)}$$

where ${\mathrm{df}}_{s}=n-1$, ${\mathrm{df}}_{t}=\tilde{k}-1$, ${\mathrm{df}}_{e}=\left(\sum_{i=1}^{n}k_{i}-1\right)-{\mathrm{df}}_{s}-{\mathrm{df}}_{t}$, *k*_*i*_ denotes the number of predator diets for the *i*th predator and $\tilde{k}=n/\sum_{i}^{n}1/k_{i}$. Note that the change to the mean squares that we suggest is not required in Lessells and Boag (1987) because, in the one‐way setting, *k* is not needed in their computation. Furthermore, when $k_{i}=k\;\text{for}\;i=1,\ldots ,n$, Equation (4) reduces to Equation (2).

Although the sums of squares can be computed using the *adonis* function, a subtlety is that the order in which the terms are entered into the model now matters, and we calculate $\tilde{\rho }$ by entering the predator factor first followed by the time factor. Note that for our example, the effect on the repeatability if time is entered first is fairly minor. The estimates $\hat{\rho }$ and $\tilde{\rho }$ can be computed using the function *comp.rep* in the QFASA R package (Stewart et al., 2021). The estimates, however, are bias‐adjusted, and the need for this modification is discussed below.

### 2.2 Interval estimation

The estimators given in Equations (2) and (4) are point estimators of *ρ*, the true or population repeatability, which we more precisely define below. Given that the estimates will vary from sample to sample and that the diets of the predators need to be estimated, confidence intervals (CIs) for *ρ* that accurately reflect these sources of error are needed. Because our framework is nonparametric, we estimate the distribution of our estimators for *ρ* using resampling methods. To motivate our CI algorithms, we begin with a discussion of the various sources of error inherent in repeated measurement of QFASA data. To simplify the discussion, we focus on the balanced setting, but we use the identical approach when there is missing data.

**FIGURE 1:**
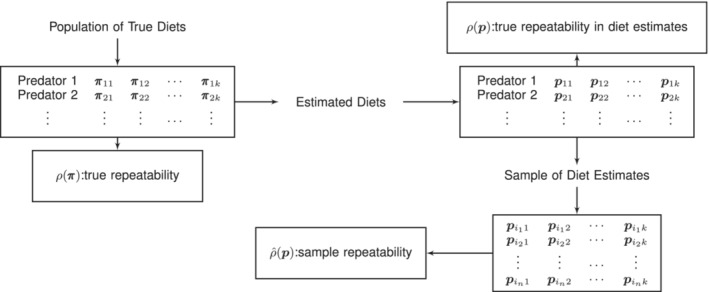
Illustration of repeatability framework and notation where $\pi_{\mathrm{ij}}$ denotes the true diet, and $p_{\mathrm{ij}}$ the corresponding QFASA diet estimate, of the *i*th predator at time *j*, i=1,…,n and j=1,…,k. The QFASA diet estimate of the *m*th predator in the sample is denoted by $p_{i_{m}j},m=1,\ldots ,n$. The notation $\rho \left(\cdot \right)$ and $\hat{\rho }\left(\cdot \right)$ is used to represent the true versus sample repeatability, respectively, and is measured by the intraclass correlation coefficient.

Given a population of predators of interest at *k* points in time, let $\pi_{11},\pi_{12},\ldots ,\pi_{1k},\pi_{21},\pi_{22},\ldots ,\pi_{2k},\ldots$ denote the actual diets of these predators. We are then interested in $\rho \left(\pi \right)$, the true repeatability in this population, which we define to be the ICC based on the actual/true diets. Since in practice the actual diets are unknown and estimated by QFASA, we define $\rho \left(p\right)$ to be the conceptual ICC of the corresponding population of QFASA diet estimates. We estimate $\rho \left(\pi \right)$ from a sample of *k* QFASA diet estimates for each of *n* predators, which we denote by $\hat{\rho }\left(p\right)$. Error in our estimator $\hat{\rho }$ arises from two sources which we refer to as (1) sampling error and (2) measurement error. The sampling error is simply the result of using a sample of *n* predators to estimate $\rho \left(\pi \right)$ rather than the entire population of predators, since another sample of *n* predators would presumably yield another estimate of $\rho \left(\pi \right)$. The second source of error, which we have termed the measurement error, can be attributed to the fact that the actual/true diets of the predators are not known but are estimated via QFASA and that the prey FA signatures, calibration coefficients, and prey fat content used in QFASA estimation are all subject to sampling variability. The distinction between the various sets of diets, as well as the associated notation, is depicted in Figure 1.

If we ignore for the moment the measurement error (that is, we assume that $\rho \left(\pi \right)=\rho \left(p\right)$), we can estimate the sample‐to‐sample variability in $\hat{\rho }\left(p\right)$ in a straightforward manner using a nonparametric bootstrap in which the predators are sampled with replacement and, for each sample of predators, the corresponding *k* diets are selected to be part of the bootstrap sample. This approach for bootstrapping clustered (albeit univariate) data was recommended in Ukoumunne et al. (2003). Specifically, for each of *R* bootstrapped samples of predator diets, we compute ${\hat{\rho }}^{*r}$, r=1,…,R and CIs based on the bootstrap distribution of the ${\hat{\rho }}^{*}$ can then be computed.

In the more realistic setting in which the QFASA diet estimates are merely estimates of the true diets, we need to account for the difference between $\rho \left(\pi \right)$ and $\rho \left(p\right)$, which we call the bias. Our approach, detailed below, for incorporating the bias involves shifting our CIs by an estimate of the bias. Note that in Stewart and Field (2011), CIs for the true diet of a predator were developed, and they also had to be shifted by an estimated amount due to a bias in the QFASA diet estimates.

To incorporate measurement error into our bootstrap procedure, we use pseudo‐predators. From the outset of QFASA, pseudo‐predators have been used in QFASA applications as a means of assessing new methodology for QFASA by allowing researchers to simulate samples of FA signatures representative of real‐life predator signatures but with specifically chosen diets. Various versions of the basic pseudo‐predator algorithms developed in Iverson et al. (2004) now exist and have been used for a variety of purposes (Bromaghin et al., 2016; Bromaghin, Budge, Thiemann, & Rode, 2017; Stewart, 2013, 2017; Stewart & Field, 2011). The core idea is to create a FA signature by sampling a prey FA library proportionately based on a given diet vector of proportions considered to be the “true” diet. The diet of the pseudo‐predator can then be estimated using QFASA, yielding a simulated diet vector.

**FIGURE 2:**
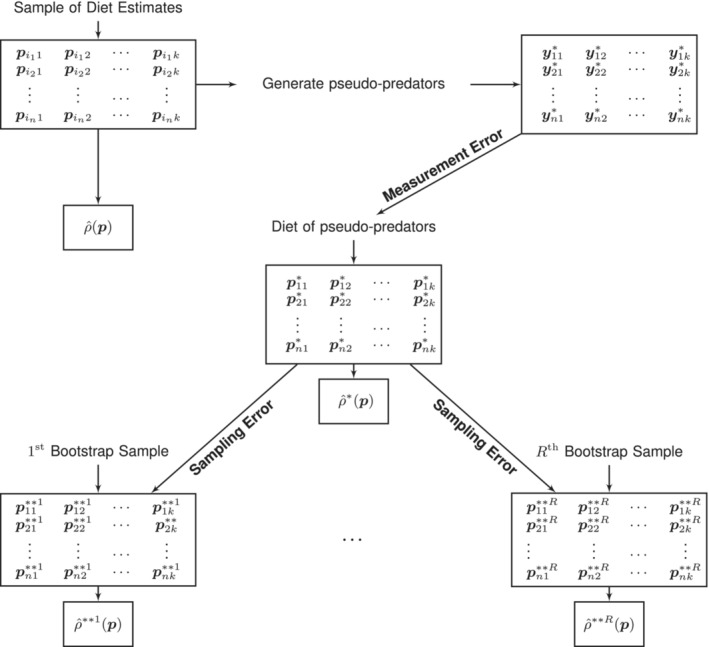
Illustration of bootstrap framework where $p_{i_{m}j}$ denotes the QFASA diet estimate of the *m*th predator in the sample, m=1…,n. The sample repeatability is denoted by $\hat{\rho }\left(p\right)$, the repeatability of the diets of the pseudo‐predators by ${\hat{\rho }}^{*}\left(p\right)$ and the repeatability in the *r*th bootstrap sample by ${\hat{\rho }}^{**r}\left(p\right)$. Repeatability is measured by the intraclass correlation coefficient and the bootstrap samples attempt to capture the sampling error. Confidence intervals are obtained by repeating the procedure *B* times.

Rather than resampling the diet estimates obtained from our original sample of predators, we propose adding measurement error by bootstrapping the estimated diets of *pseudo‐predators*, where their diets are determined from the diet estimates of our original sample. More specifically, for *n* sampled predators with diet estimates denoted by $p_{i_{1}1},\ldots ,p_{i_{1}k}\ldots ,p_{i_{n}1}\ldots ,p_{i_{n}k}$ in Figure 1, we generate corresponding pseudo‐predators depicted as $y_{11}^{*},\ldots ,y_{1k}^{*}\ldots ,y_{n1}^{*}\ldots ,y_{\mathrm{nk}}^{*}$ in Figure 2. The estimated diets of the pseudo‐predators, $p_{11}^{*},\ldots ,p_{1k}^{*}\ldots ,p_{n1}^{*}\ldots ,p_{\mathrm{nk}}^{*}$, are then bootstrapped *R* times and the entire procedure, as shown in Figure 2, is repeated *B* times, yielding estimates ${\hat{\rho }}^{**\mathrm{br}}\left(p\right)$, r=1,…,R, and b=1,…,B. Note that *b* indexes the number of generated samples of pseudo‐predators.

Using the bootstrap samples, we compute the bootstrap studentized *T* and BCa intervals in Davison and Hinkley (1997). In general, for a parameter of interest, say θ, being estimated by *T* with variance *V*, the bootstrap studentized *T* intervals use a bootstrap approximation to the distribution of $Z=\left(T-\theta \right)/V^{1/2}$ instead of the usual normal approximation. Confidence limits then follow and are analogous to the Student‐*t* confidence limits for a population mean. Note that an estimate of the standard error ($V^{1/2}$) is needed and we use the “jackknife” function in the bootstrap R package (Tibshirani, 2019) to accomplish this. Percentile methods offer an alternative bootstrapped‐base approach to interval estimation and the BCa intervals, in particular, incorporate bias and skewness correction factors. In Rizzo (2012), BCa is referred to as the “better bootstrap confidence interval,” and we have therefore chosen to investigate these intervals in addition to the simpler *T* intervals. The bias and acceleration factors were estimated as in Rizzo (2012), with jackknife replicates being used to estimate the acceleration factor. Note that in Figure 2, the bootstrap confidence intervals estimate $\rho^{*}\left(p\right)$, the population version of ${\hat{\rho }}^{*}\left(p\right)$. We need to therefore account for the difference between $\rho^{*}\left(p\right)$ and $\rho \left(p\right)$, and subsequently, the difference between $\rho \left(p\right)$ and $\rho \left(\pi \right)$, where $\rho \left(\pi \right)$ is ultimately our parameter of interest. Let $d_{1}=\rho^{*}\left(p\right)-\rho \left(p\right)$ and $d_{2}=\rho \left(p\right)-\rho \left(\pi \right)$. We estimate *d*_1_ by comparing the mean of the *B* estimates, ${\hat{\rho }}^{*b}\left(p\right)$, with $\hat{\rho }\left(p\right)$. We also make the assumption that $d_{1}=d_{2}=d$ so our estimate of the common bias *d* is

(5) $$\hat{d}=\frac{1}{B}\sum_{b=1}^{B}{\hat{\rho }}^{*b}\left(p\right)-\hat{\rho }\left(p\right).$$

Note that this is analogous to the bootstrap estimate of bias (Davison & Hinkley, 1997). Our total bias is then 2*d*_,_ which is estimated by $2\hat{d}$, and our CIs are shifted (the bias is subtracted from the end points) by this amount. In our applications, this bias was found to be negative (see Section 3) so by subtracting the bias, we are, in effect, adding its absolute value to the end points of the CIs.

It is important to note that if $\hat{\rho }\left(p\right)$ is reported on its own (that is, without the accompanying interval), we require only an estimate of *d*_2_, the difference between $\rho \left(p\right)$ and $\rho \left(\pi \right)$, and so, our intervals are shifted by $\hat{d}$ in Equation (5). We recommend this adjustment if, as is the usual case, the diets are estimated with error.

In summary, the bias‐adjusted estimate of $\hat{\rho }\left(p\right)$ is $\hat{\rho }\left(p\right)-\hat{d}$, and the corrected bootstrap CIs are $\left(L_{\text{boot}}-2\hat{d},U_{\text{boot}}-2\hat{d}\right)$, where $L_{\text{boot}}$ and $U_{\text{boot}}$ denote the lower and upper CI bootstrap limits, respectively. The bias‐corrected CIs are computed in the QFASA R package (Stewart et al., 2021) through the function *comp.rep* if the parameter *CI* is set to “TRUE.”

### 2.3 Simulation study

We applied a simulation study to assess the accuracy and precision of our proposed measure of repeatability in the context of QFASA diet estimates, where our measure estimates the repeatability of a sample of n×k diet estimates from a conceptual population of diets with true repeatability $\rho \left(\pi \right)$. We created a simulated population of diets (as described below) by generating five different large “grids” of population diets corresponding to five different values of $\rho \left(\pi \right)$, where each grid is similar to what is depicted in the upper left‐hand corner of Figure 1, and sampled repeatedly from each grid. Then, to simulate observing QFASA diet estimates rather than the actual diets, we generated pseudo‐predators from each sample of selected diets and computed their QFASA diet estimates. Finally, we obtained 95% CIs for $\rho \left(\pi \right)$ based on the diet estimates, using the methods in Section 2.2, and computed (1) the proportion of time the intervals included the true repeatability ($\rho \left(\pi \right)$) or the associated *coverage probability* and (2) the average length (or width) of the intervals, where the length of an individual interval is simply the difference between the upper limit and lower limit. Coverage probabilities near 0.95 are desired, as well as CIs that are not too wide to be useful in practice since shorter CIs reflect more precise knowledge about our parameter of interest.

To create the grids of population diets, each with an associated value of $\rho =\rho \left(\pi \right)$, we first took an average of the FALL/WINTER grey seal QFASA diet estimates described in Section 2.4 and then modified this average diet systematically. Specifically, the average was transformed using the isometric log‐ratio (ilr) transformation (Egozcue et al., 2003), a commonly used and recommended transformation for compositional data based on its mathematical properties, and a measure of variability was also obtained from the ilr transformed diet estimates. To obtain 5 grids of diets with different corresponding values of $\rho \left(\pi \right)$, we modified the transformed base diet through the addition of 1000 chosen “row effects,” 2 “column effects” (corresponding to k=2), and normal random error. The 2000 diet estimates were then transformed back to compositions. The row effect was generated from a multivariate normal distribution with mean given by a vector of zeros and the covariance matrix given by a diagonal matrix with diagonal elements obtained from the estimated variances in the real‐life diet estimates, as previously described. Since we were interested in 5 increasing values of *ρ* with the minimum value near 0 and the maximum value near 1, for each of the 1000 diets, the column effect was ± a constant times the mean diet vector. The repeatability was computed using Equation (2) for each grid of 2000 diets and the resulting values of *ρ* were ρ=0.051,0.261,0.510,0.709, and 0.947.

**FIGURE 3:**
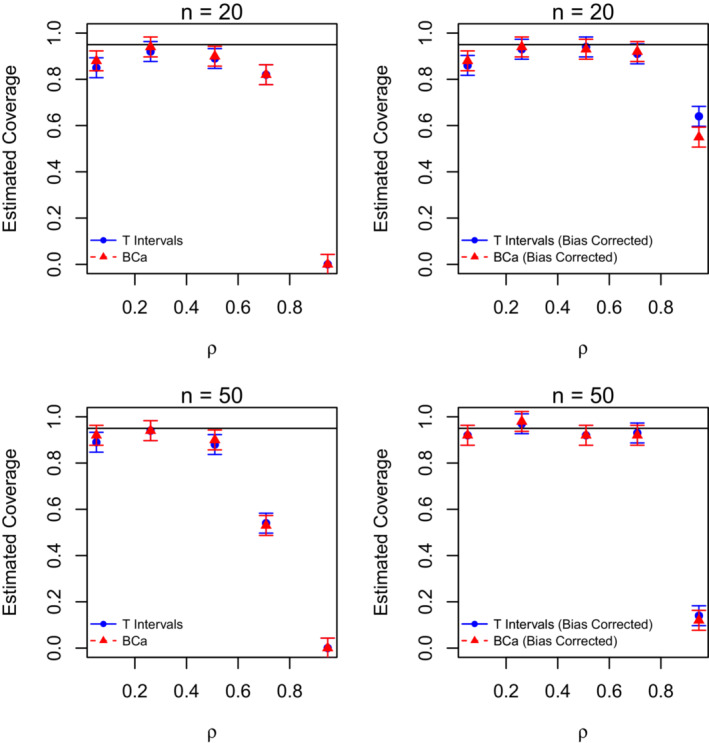
Comparison of estimated coverage probabilities (and corresponding margin of errors for coverage) for 95% bootstrap *T* and BCa confidence intervals, with and without bias correction for two sample sizes (20 and 50) and two simulated time periods corresponding to various values of repeatability denoted by *ρ*.

The algorithm for yielding a single confidence interval is computationally demanding; therefore, there were practical limitations on *n*, *B*, *R*, and *k*, as well as the number of simulations that could be run. To this end, we ran our simulations in parallel with 5 cores and examined only modest values of *n* (n=20 or n=50; Figure 3), with k=2, B=100, and R=100. We surmise that results would improve with increasing *n*, but as the computational burden also increases with sample size, it was not feasible to examine large values. We were also limited in the number of total simulations we could reasonably run (in particular for n=50), and this was set to 100 for both sample sizes, resulting in coverage probabilities with an associated margin of error of approximately 4%.

### 2.4 Quantifying grey seal diets

#### 2.4.1 Seal samples

Full‐depth blubber biopsies were collected between 1993 and 2015 from 220 adult, free‐ranging, grey seals (90 males, 130 females) on Sable Island, NS, Canada (43°55′N, 60°00′W) following the methods described in Beck et al. (2007). Samples were collected during the molt (May–June, SPRING), in September–October (FALL), or during the annual breeding season (December–January, WINTER) as part of studies examining diet, energetics, foraging distribution, and behavior (Austin et al., 2006; Beck et al., 2007; Breed et al., 2006; Lang et al., 2009, 2011; Lidgard et al., 2003, 2020; Mellish et al., 1999; Noren et al., 2005). Individuals were either sampled at two different periods within the same calendar year (SPRING and subsequent WINTER or FALL and subsequent WINTER, Table 1) or in the same period (WINTER) over multiple years (Table 2). In the latter case, note that from Table 2, there are several missing values. Prey FAs are deposited in blubber over time (Cooper, 2004; Iverson et al., 2004) such that the FA composition of grey seal blubber represents the integrated diet over the preceding two to three months. Thus, samples collected during the SPRING, FALL, and WINTER periods described above will reflect the integration of the diet consumed in spring, late summer, and fall/early winter, respectively. Details of the sample processing methods can be found in Budge et al. (2006). Note that over the course of lactation, female grey seals do not mobilize blubber FAs in a uniform manner (Arriola et al., 2013), therefore, all blubber samples collected from lactating females during the breeding season (WINTER) were collected prior to day 6 postpartum. All procedures used on study animals were conducted in accordance with the legal requirements of the Canadian Council on Animal Care following the Guidelines on the Care and Use of Wildlife. All procedures were approved by Dalhousie University's Committee on Laboratory Animals and by Fisheries and Oceans Canada's animal care committee.

**TABLE 1:** Number of adult grey seal males and females sampled twice within the same calendar year by year.

Year	SPRING/WINTER		FALL/WINTER	
	Male	Female	Male	Female
1993	4	3		
1994	1	4		
1995	4	5	1	3
1996	9	7	5	5
1997	7	5	3	2
1998	7	2	4	
1999	3	3	6	4
2000			4	4
2001				5
2004	6	3		
2009			7	9
2010			6	8
2011		15		
2012	5	9		2
2013	4	5		
2014	4	5		
Total	54	66	36	42

*Note*: SPRING/WINTER, individuals sampled during the molt in May–June (SPRING) and then again during the subsequent breeding season (December–January, WINTER). FALL/WINTER, individuals sampled in September–October (FALL) and then again during the subsequent breeding season (WINTER). Individual seals appear only once in the data set (i.e., either sampled at SPRING/WINTER or FALL/WINTER) across all years.

**TABLE 2:** Number of samples for each of 24 adult grey seal females sampled across multiple years during the breeding season (December–January, WINTER).

Seal ID	Year							Total
	1999	2000	2001	2002	2003	2004	2006	
Hg12	·			·				2
Hg23	·	·	·	·				4
Hg32		·		·				2
Hg112	·	·	·					3
Hg132	·		·	·		·		4
Hg501	·		·			·		3
Hg505	·	·		·	·			4
Hg825	·	·		·				3
Hg2675	·	·	·	·				4
Hg3250	·	·		·				3
Hg3263	·		·					2
Hg3625	·		·		·			3
Hg3817	·	·	·	·				4
Hg3994	·	·	·					3
Hg4374	·		·		·			3
Hg4377	·	·			·			3
Hg4388	·	·		·				3
Hg4391	·	·	·	·		·		5
Hg4393	·	·	·	·				4
Hg4404	·	·		·				3
Hg4489	·		·	·	·		·	5
Hg4491	·	·	·	·	·			5
Hg4735	·		·	·	·			4
Hg6035			·	·				2

#### 2.4.2 Prey library

For the QFASA estimation of seal diets (see QFASA Diet Estimates below) we used a prey database (“library”) comprised of 1735 individuals FA signatures from 21 species of fish and invertebrates that were collected within the main foraging range of the Sable Island grey seals (Northwest Atlantic Fisheries Organization 4 Subarea, excluding the Gulf of St Lawrence estuary). The 21 prey species included in the library (Table 3) are those known to be eaten by grey seals based on previous stomach content and fecal analyses (Bowen et al., 1993; Bowen & Harrison, 1994) or prey that was reasonably abundant and found at depths at which grey seals are known to forage (Beck et al., 2003a, 2003b). Details of prey collection and processing can be found in Budge et al. (2002).

**TABLE 3:** Prey species (“prey library”) used in QFASA to estimate the diet composition of adult grey seals.

	Common name	Scientific name	Cluster	Modeling Set 1		Modeling Set 2	
				*n*	Lipid (%)	*n*	Lipid (%)
Forage Fish	Atlantic butterfish	*Peprilus triacanthus*	March	49	12.3		
			July–September	26	8.1	26	8.1
	Capelin	*Mallotus villosus*	March–May	135	4.7		
			July	27	7.7	27	7.7
			September			21	9.7
	Atlantic herring	*Clupea harengus*	March	108	2.3		
			July–September	121	10.1	121	10.1
	Atlantic mackerel	*Scomber scombrus*		32	5.1	32	5.1
	Northern sand lance	*Ammodytes dubius*		148	5.3	148	5.3
	Snake blenny	*Lumpenus lumpretaeformis*		18	2.4	18	2.4
Gadids	Atlantic cod	*Gadus morhua*		109	2.5	109	2.5
	Pollock	*Pollachius virens*	Group 1	35	1.9	35	1.9
			Group 2	18	3.6	18	3.6
	Silver hake	*Merluccius bilinearis*		58	1.6	58	1.6
	White hake	*Urophycis tenuis*		80	1.3	80	1.3
Flatfish	American plaice	*Hippoglossoides platessoides*	Small (<25 cm)	67	2.9	67	2.9
			Large (>25 cm)	67	1.8	67	1.8
	Winter flounder	*Pseudopleuronectes americanus*		50	2.0	50	2.0
	Witch flounder	*Glyptocephalus cynoglossus*		24	1.9	24	1.9
	Yellowtail flounder	*Limanda ferruginea*		156	2.2	156	2.2
Skates	Smooth skate	*Malacoraja senta*		33	2.5	33	2.5
	Thorny skate	*Amblyraja radiata*		83	2.6	83	2.6
	Winter skate	*Leucoraja ocellata*		40	1.5	40	1.5
Other	Redfish	*Sebastes sp*.		54	7.1	54	7.1
	Longhorn sculpin	*Myoxocephalus octodecemspinosus*	March–April	45	2.1		
			September			25	4.0
	Sea raven	*Hemitripterus americanus*		71	2.0	71	2.0
Invertebrates	Northern shortfin squid	*Illex illecebrosus*		35	3.0	35	3.0
				1689		1398	

Following an exploratory analysis to determine whether the FA signatures of the selected prey contained any hidden structure (see Bromaghin, Budge, & Thiemann, 2017) some prey species within the set were subdivided into smaller clusters prior to estimating seal diets (Table 3). American plaice (*Hippoglossoides platessoides*) were separated into two clusters based on size (small, ≤25 cm, and large, >25 cm). Pollock (*Pollachius viren*s) were separated into two clusters based on observed substructure among the FA signatures although the proximate cause for the substructure was unclear (there was no relationship to differences in size, season, or collection location). Substructure in the FA signatures based on seasonal variation (collection months) was found in four species (Atlantic butterfish, *Peprilus triacanthus*; Atlantic herring, *Clupea harengus*; capelin, *Mallotus villosus*; and longhorn sculpin, *Myoxocephalus octodecemspinosus*). Based on the identified substructure for these four species, the FA signatures from individual prey collected in March, April, and May were used to model the diets of seals sampled in SPRING (see Table 3, Modeling Set 1) while the FA signatures of individual prey collected from July onward were used to model the diets of seals sampled in FALL and WINTER (Table 3, Modeling Set 2).

#### 2.4.3 QFASA diet estimates

The diet of each grey seal at each sampling point was estimated using QFASA following the methods of Iverson et al. (2004). The diets were modeled using the calibration coefficients developed for grey seals (see Iverson et al., 2004). We used the “Dietary” FA subset, as defined in Iverson et al. (2004), excluding 16:3n‐1, 16:4n‐3, 22:n‐6, and the 20:1 isomers. The FAs 16:3n‐1, 16:4n‐3, and 22:n‐6 were not identified across all samples in the data set and were, therefore, removed from all analyses. The calibration coefficients for the 20:1 isomers for grey seals (and other phocid seals) are very small, which can create calibrated predator FA signatures with values for these isomers, which are outside the range of values observed in the potential prey resulting in estimation issues (see Bromaghin et al., 2015). For the six prey species whose FA signatures were subdivided into clusters prior to QFASA modeling (see Section 2.4.2), the QFASA diet estimates for the clusters (where applicable) were summed to give a single proportion for that prey species prior to calculating repeatability.

The diet estimates for the individuals sampled at two different periods within the same calendar year (SPRING/WINTER or FALL/WINTER, Table 1) are shown in Tables S1 and S2, respectively. Individual seals appear only once in the full data set (i.e., either sampled at SPRING and WINTER or FALL and WINTER) across all years. Diet estimates for the 24 females sampled in WINTER over multiple years (Table 2) are shown in Table S3. Given that the diet estimates are of length 21 (as there are 21 potential prey species in the diet), we consider n=24 to be a relatively small sample size.

## 3 RESULTS

Estimated coverage probabilities for 95% confidence intervals for *ρ*, along with error bars reflecting the 4% margin of errors, are given in Figure 3. The first row corresponds to n=20 while the second row pertains to n=50. The coverage with and without the bias correction is illustrated for comparison purposes. Recall that the bias adjustment is needed to account for the measurement error due to using QFASA diet estimates. The solid line at 0.95 indicates the target coverage and, after being corrected for measurement error, all intervals appear to yield reasonably high coverage except when *ρ* is extremely large (that is, for ρ=0.95). Note that when ρ=0.71, the bias correction greatly improves the coverage probability in all cases, but the improvement is insufficient for ρ=0.95. While we may potentially conclude that estimating repeatability when temporal consistency is very high may be problematic, repeatability as high as 0.95 seems unlikely in practice. Furthermore, while the coverage of the intervals is lower than the target coverage of 0.95, when *ρ* is large, the upper bound of the intervals is typically near this value. Specifically, on average, the upper limit of both the *T* and BCa CIs was 0.95 for n=20 and 0.93 for n=50. The bias‐corrected *T* and BCa intervals are similar for both sample sizes and the coverage at n=50 is generally either roughly the same or larger than that at n=20, except for when ρ=0.95.

The average width of the intervals is compared in Table 4. The widths suggest that confidence intervals appear to be largest for values of *ρ* near 0.5 and increasing the sample size noticeably reduced the length of the intervals on average. The *T* and BCa intervals appear to be of similar lengths.

**TABLE 4:** Comparison of average confidence interval lengths for 95% bootstrap *T* and BCa confidence intervals for two sample sizes (20 and 50) and two simulated time periods corresponding to various values of repeatability denoted by *ρ*.

	ρ=0.05		ρ=0.26		ρ=0.51		ρ=0.71		ρ=0.95	
	n=20	n=50	n=20	n=50	n=20	n=50	n=20	n=50	n=20	n=50
*T*	.141	.080	.267	.158	.287	.184	.249	.169	.123	.089
BCa	.132	.080	.274	.161	.289	.187	.249	.174	.122	.093

**FIGURE 4:**
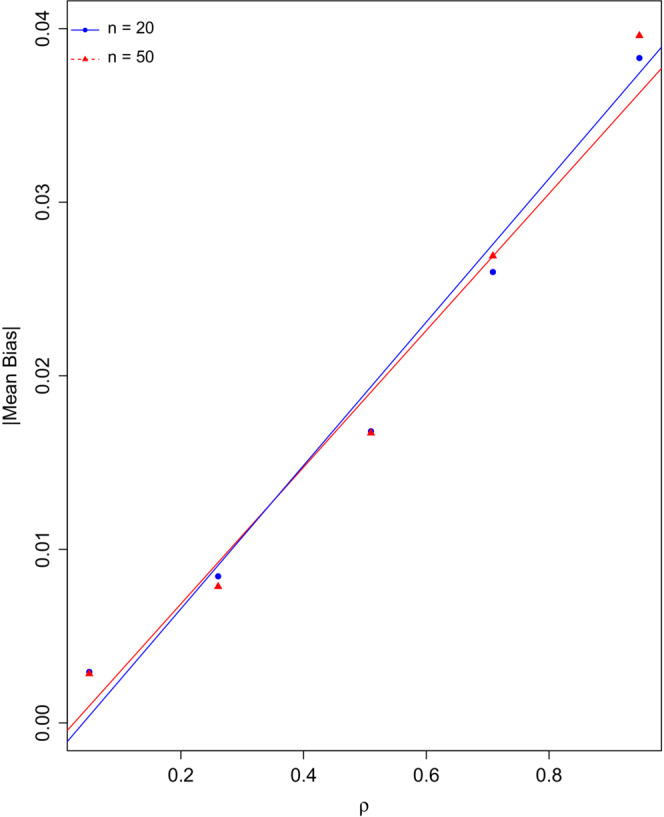
Plot of the magnitude of the average estimated bias in the repeatability estimates over the simulations versus the true values of repeatability denoted by *ρ*.

In Figure 4 the absolute value of the average estimated bias (see Equation 5) over the 100 simulated samples is plotted against *ρ*. It is clear that the bias (in magnitude) increases as the true value of *ρ* increases and in a seemingly linear manner. This bias (resulting from measurement error) is not affected by the sample size.

### 3.1 Grey seal diets

We applied our proposed measures of repeatability for compositional data for both balanced and unbalanced designs to the grey seal QFASA diet estimates. The diet estimates for individuals sampled at two different periods within the same calendar year (SPRING/WINTER, Table S1; FALL/WINTER, Table S2) were used to estimate repeatability (balanced design) across seasons within years (seasonal repeatability). The diet estimates for the 24 females sampled in WINTER over multiple years (Table S3) were used to estimate repeatability (unbalanced design) across years (interannual repeatability).

#### 3.1.1 Seasonal repeatability in diet

Recall that to obtain CIs for repeatability, we generate pseudo‐predators by setting the “true” diet in the pseudo‐predator algorithm to be the QFASA diet estimates. Pseudo‐predators are then generated by sampling proportionately from an appropriate prey database. For repeatability across the SPRING/WINTER seal diets, we generated pseudo‐predators and obtained CIs using both the prey database used to estimate SPRING diets (Set 1, Table 3) and the prey database used to estimate the FALL and WINTER diets (Set 2, Table 3) in order to determine how the choice of prey database affects the estimates of CIs. For repeatability across the FALL/WINTER diets, only the prey set used to estimate FALL and WINTER diets (Set 2, Table 3) was used to generate the pseudo‐predators. The point estimates of repeatability and CIs (all of which have been adjusted by an estimate of bias) are presented in Table 5.

**TABLE 5:** Estimated repeatability ($\hat{\rho }$) and 95% *T* and BCa confidence intervals for the true seasonal repeatability in the diets of grey seals.

Seasons	Prey set	Type	*n*	$\hat{\rho }$	95% CI
Spring/Winter	Set 1	T	120	0.293	(0.235, 0.355)
		BCa	120	.293	(0.235, 0.356)
Spring/Winter	Set 2	T	120	.293	(0.237, 0.356)
		BCa	120	.293	(0.237, 0.357)
Fall/Winter	Set 2	T	78	.617	(0.544, 0.686)
		BCa	78	.617	(0.542, 0.686)

Bootstrap parameters were set to B=50 and R=100 for both the SPRING/WINTER and FALL/WINTER repeatability to accommodate the large sample sizes. Specifically, as the algorithm requires distances between all pairs of observations to be calculated for each bootstrap sample, the larger the sample size, the slower it is to obtain the intervals. Note that with these settings, CIs are based on 50×100=5000 bootstrap samples.

The repeatability across the SPRING/WINTER diet estimates was low indicating little temporal consistency in the diets of individuals between spring and the subsequent fall/early winter period. For the SPRING/WINTER comparison, the T and BCa intervals were almost identical regardless of the choice of prey modeling set (Table 5). In contrast to the repeatability for SPRING/WINTER, the repeatability across the FALL/WINTER diets was high indicating that there is a temporal consistency in the diets of individuals over the period from late summer to fall/early winter.

#### 3.1.2 Interannual repeatability in diet

Bootstrap *T* and BCa 95% confidence intervals were computed for the QFASA diet estimates of the 24 individual female grey seals sampled in 2–5 different WINTER seasons in Table 2. As *k* is not fixed, this is an example of an unbalanced setting with missing values and, thus, Equation (4) was used to calculate repeatability. Results are given in Table 6. The prey set used to estimate FALL and WINTER diets (Modeling Set 1, Table 3) was used to obtain the CIs.

**TABLE 6:** Estimated repeatability ($\hat{\rho }$) and 95% *T* and BCa confidence intervals for the true interannual repeatability in the diets of female grey seals between 1999 and 2006.

*n*	$\hat{\rho }$	Type	95% CI
24	.725	*T*	(0.634, 0.831)
		BCa	(0.633, 0.825)

As before, the results are very similar for the *T* and BCa intervals. The bias was large in this example ($\hat{\rho }\left(p\right)$ was shifted upwards by 0.247) and the intervals were shifted by double this amount due to *ρ* being large and, we surmise, more repeated measurements. The interannual repeatability was high indicating that there was a temporal consistency across years in the diets of these individuals during the fall/early winter period prior to the start of the breeding season.

## 4 DISCUSSION

Here, we propose a statistical approach for assessing the temporal consistency in compositional diet estimates using a measure of repeatability for the multivariate setting. In contrast to indices that have previously been used to examine individual consistency in concurrent, compositional diet estimates over different time scales (e.g., Estes et al., 2003; Thiemann et al., 2011), our approach provides a means of simultaneously incorporating the variance in resource use associated with both the individual and the population, providing an empirical measure of the extent to which compositional diet estimates are characteristic of individuals. Our measure can be applied to situations in which the sample sizes are small relative to the number of potential prey species in the diet and we further developed an extension that can accommodate unbalanced designs (missing values). While, in general, missing observations are statistically challenging to deal with, they are common in real‐life data sets and this extension, therefore, allows our methods to be more widely applied. A critical component of our approach is the use of an appropriate distance measure. By choosing the chi‐square distance measure we do not have to modify or condition on  the zeros in the diet estimates, as has been done in the literature with problematic zeros in compositional data (Palarea‐Albaladejo & Martín‐Fernández, 2008, 2013; Stewart & Field, 2011).

When the diet of predators is estimated using QFASA, we found that our estimate of repeatability may be biased due to the QFASA diet estimates being highly variable estimates of the true unknown diets. More specifically, our estimate of repeatability tends to be consistently smaller than *ρ* and the magnitude of the bias increases with increasing values of *ρ*. Our CI methods adjust for this bias and, even when *n* is small relative to the dimension of the diet estimates, appear to perform well. Our measure of repeatability can easily be applied to compositional diet estimates derived from other methods such as stomach content or scat analysis, for example, as well as to other fields of applications where compositional measurements over time have been collected. In applications beyond QFASA, CIs that incorporate sampling error could be developed in a relatively straightforward analogous manner; however, our approach for incorporating measurement error into the CI algorithm is unique to QFASA diet estimates and this component of the CI methodology cannot be extended to other applications.

We applied our proposed measure of repeatability to QFASA diet estimates of free‐ranging grey seals in the Northwest Atlantic. The results of the seasonal comparisons indicate that there is low temporal consistency in the diets of individual grey seals between the spring and fall/early winter but a relatively high level of temporal consistency in individual diets in the period between late summer and fall/early winter of a given year (Table 5). These results are consistent with seasonal changes in grey seal diets observed in cross‐sectional comparisons of QFASA diet estimates (Beck et al., 2007) and with the seasonal shifts observed in stable isotope values across grey seal tissues (Hernandez et al., 2019). Although our results are consistent with previously observed seasonal shifts in grey seal diets, the present analysis did not consider the potential influence of factors such as sex or changes in population abundance on the patterns of seasonal repeatability. The diets of male and female grey seals are known to differ with males typically having more diverse diets than females (Beck et al., 2007; Tucker & Iverson, 2007). In addition, the diet estimates used in the present study cover a period of significant growth for this population (den Heyer et al., 2021), and thus, potential changes in the level of intraspecific competition may be occurring which is predicted to influence the degree of individual specialization (Araújo et al., 2011). Future analyses examining the influence of these factors on the levels of seasonal repeatability will be required to more fully understand the time scales over which individual consistency in the diet is occurring in grey seals.

The high level of repeatability in the QFASA diet estimates of individual females during winter sampling across years (Table 6) is also in keeping with the individual consistency observed in the isotopic niches of grey seals (Hernandez et al., 2019). Our results indicate that there was a strong individual consistency in the fall/early winter diets of the females sampled here. However, a more detailed analysis of the species composition of the individual diets will be required to determine whether the high level of repeatability across years is due to consistent generalist diets or whether individual females may be specializing in a small range of prey types in the period leading up to the breeding season.

A downside of our measure of repeatability is that it can be slow to compute, particularly when the sample size, number of time points, or number of prey species is large. Since the CI procedure is bootstrapped‐based and requires computing QFASA diet estimates, it is extremely computationally intensive. If CIs are desired, the bootstrapping could potentially be done in parallel to speed up computations. While the computational burden limited how extensively we could examine our proposed method through simulations, the real‐life data sets provided additional validation and information on the precision (as reflected in the length of the CIs) likely to be observed in practice.

The conclusions drawn regarding repeatability in the real‐life data, as well as our simulation study results, suggest that our novel measure of repeatability is useful and capable of handling complex compositional data sets, such as those comprising of diet estimates. Given the importance of understanding how predator diets vary over time and space, our method may find broad application.

## AUTHOR CONTRIBUTIONS

**Connie Stewart:** Conceptualization (equal); formal analysis (lead); methodology (lead); software (lead); writing – original draft (equal); writing – review and editing (equal). **Shelley L. C. Lang:** Conceptualization (equal); data curation (lead); writing – original draft (equal); writing – review and editing (equal). **Sara Iverson:** Conceptualization (equal); resources (equal); writing – review and editing (equal). **W. Don Bowen:** Conceptualization (equal); resources (equal); writing – review and editing (equal).

## CONFLICT OF INTEREST

The authors declare that they have no conflict of interest.

## Supporting information


Table S1

Table S2

Table S3

## Data Availability

Data available in Supporting Information (Tables [Link], [Link]).
