# Supplementary material for: Measuring repeatability of compositional diet estimates: An example using quantitative fatty acid signature analysis
Source: Ecol Evol. 2022 Oct 27;12(10):e9428. doi: 10.1002/ece3.9428 (PMC9608821; doi:10.1002/ece3.9428)
Supplement: Supplementary file 1 — Table S1 [file ECE3-12-e9428-s001.pdf]

Table A1. QFASA diet estimates for 120 adult grey seals (54 males, 66 females) sampled during the moult in May-June (SPRING) and then again during the subsequent breeding season (December-January, WINTER). M, male; F, female.

Seal ID	Sex	Year	Season	Atlantic Butterfish	Capelin	Atlantic Herring	Atlantic Mackerel	Northern Sandlance	Snake Blenny	Atlantic Cod	Pollock	Silver Hake	White Hake	American Plaice	Winter Flounder	Witch Flounder	Yellowtail Flounder	Smooth Skate	Thorny Skate	Winter Skate	Redfish	Longhorn Sculpin	Sea Raven	Northern Shortfin Squid
Hg616	M	1993	SPRING	0.7	20.6	22.4							22.3							13.8	20.1			
			WINTER							36.4	3.1		31.6								26.7			2.3
Hg630	M	1993	SPRING	4.2		48.9							30.7								16.1			
			WINTER		3.4					29.0	22.7		33.9								11.0			
Hg2643	M	1993	SPRING		12.0	17.1										19.6	22.5				28.7			
			WINTER		0.8					27.9	12.0		25.1			10.8					23.5			
Hg3602	M	1993	SPRING	1.7	5.0	39.3							6.2							18.8	29.0			
			WINTER		3.8					59.1			11.1								25.4			0.7
Hg3604	M	1994	SPRING	1.4	11.2	10.6					38.2		6.3							19.6	12.7			
			WINTER	0.5	2.3						34.2		50.2								12.9			
Hg2602	M	1995	SPRING	2.8		41.4		0.5					15.4		1.3						38.6			
			WINTER		5.2			37.5							2.2						55.1			
Hg2603	M	1995	SPRING	2.2	27.8	33.5														21.1	15.4			
			WINTER		1.3			23.8		30.3	20.6			4.9							18.1	1.0		
Hg2644	M	1995	SPRING	0.1		4.7		7.4		27.4	1.7		21.6				14.9				22.3			
			WINTER		0.5			1.1		35.5	18.6				2.6		13.0				28.8			
Hg2646	M	1995	SPRING				0.1				50.7				6.2		3.2		6.0	20.9	12.8			
			WINTER		5.0					13.7	56.4				7.6				9.6	1.1	6.6			
Hg2996	M	1996	SPRING	0.6	3.9								62.0			8.4					25.1			
			WINTER		2.3				9.7	58.2							10.3				19.5	0.0		
Hg3015	M	1996	SPRING	5.2	16.0	50.2															28.6			
			WINTER					15.2		33.5	29.7				3.0						18.7			
Hg3031	M	1996	SPRING	2.2							13.5		43.1				10.3			0.1	30.9			
			WINTER		2.7			6.0		30.3	2.4						31.6				27.0			
Hg3050	M	1996	SPRING	3.1	9.9	14.3					35.9		24.5							3.9	8.4			
			WINTER					24.1		42.3	17.6		6.4	2.7							6.9			
Hg3058	M	1996	SPRING	1.7	35.5	27.5					4.6									18.5	12.2			
			WINTER					6.0		26.1	43.3			6.1	2.2						16.4			
Hg3061	M	1996	SPRING	2.4	2.2	15.7							21.0								58.8			
			WINTER		0.1			7.7	3.2	34.0					4.4						50.6			
Hg3065	M	1996	SPRING	2.1	25.6	9.5					41.5									14.5	6.9			
			WINTER		2.1			8.8		28.4	25.1		26.9								8.8			
Hg3079	M	1996	SPRING	2.2	8.3	0.0					58.4		18.4							1.2	11.5			
			WINTER					26.9		16.6	10.1		34.7								11.7			
Hg3081	M	1996	SPRING	2.1	14.6	13.7					34.2		24.0							4.0	7.4			
			WINTER		2.5			5.8		46.7	26.7		7.3								10.1			1.0
Hg617	M	1997	SPRING	4.3	7.9	36.6					9.3		26.4								15.5			
			WINTER					9.7		45.8	26.8										17.7			
Hg3626	M	1997	SPRING	2.0	42.8	26.5														12.5	16.2			
			WINTER		0.7			4.3		48.6	15.8		14.5								15.6			0.5
Hg3644	M	1997	SPRING	0.1	1.5																98.4			
			WINTER						10.2												89.8			
Hg3660	M	1997	SPRING	3.6	2.7	54.1															39.6			
			WINTER		0.4			19.5		34.8											45.3			
Hg3662	M	1997	SPRING	2.4		16.0							50.2								31.4			
			WINTER		9.4			0.5		33.9											30.5	25.7		
Hg3666	M	1997	SPRING	2.0	28.7						23.9		8.3							31.7	5.5			
			WINTER					1.2		37.7	30.2		14.3								16.6			
Hg3667	M	1997	SPRING			7.2				20.8	4.4			0.1			18.5				49.0			
			WINTER							25.1	15.4						15.4				44.1			
Hg764	M	1998	SPRING	0.5							50.3		31.8							6.6	10.9			
			WINTER		0.3			10.7	5.3	10.4	59.1			11.6							2.6			
Hg841	M	1998	SPRING	6.2	18.3	41.2							5.6								28.8			
			WINTER					5.2	3.4	27.7	42.7				0.6		4.2				16.3			
Hg2798	M	1998	SPRING	2.3							38.0		35.8							6.2	17.7			
			WINTER		0.7			16.0	6.0	23.6	41.9			4.1	1.2						6.4			
Hg4268	M	1998	SPRING	2.3		14.2					32.0		27.3								24.1			
			WINTER						4.5	36.7	33.7		11.1	0.7	3.2		0.1				10.1			
Hg4272	M	1998	SPRING	3.4	16.2	17.3							13.3								49.9			

			WINTER						3.1	14.6	14.7		6.0		7.2		2.9				51.4			
Hg4274	M	1998	SPRING	0.9	7.7						28.8		30.1							16.6	15.8			
			WINTER						18.9	10.0	42.3		6.8								22.1			
Hg4294	M	1998	SPRING	3.1	23.4	30.6							12.8								30.1			
			WINTER		6.1						15.1		70.0								8.9			
Hg3066	M	1999	SPRING	0.8				9.1			25.5		15.0	3.1	10.5						36.2			
			WINTER							63.3	0.3										36.3			
Hg5099	M	1999	SPRING		3.3			19.0		34.2				13.3	7.1						23.2			
			WINTER		2.6			23.9		14.3											59.1			
Hg5100	M	1999	SPRING	2.9		11.0					16.9		44.7								24.5			
			WINTER		0.0			13.0		52.9											34.1			
Hg7389	M	2004	SPRING	1.4	21.1	0.6														42.7	34.2			
			WINTER					45.5		23.7	1.3										29.4			
Hg7390	M	2004	SPRING	1.2	34.5	3.6														36.3	24.5			
			WINTER		8.6																91.4			
Hg7392	M	2004	SPRING				2.1				33.5					3.6	0.7		7.3	42.8	10.1			
			WINTER					17.2			11.4		39.5							13.5	18.4			
Hg7393	M	2004	SPRING	2.2	9.6	18.4					42.6									7.1	20.1			
			WINTER					8.4		0.5	10.6		37.1	14.5							28.9			
Hg7394	M	2004	SPRING	1.6	5.0	28.5							27.5							1.4	36.1			
			WINTER					23.8		6.8	19.8		1.6								48.1			
Hg7395	M	2004	SPRING	0.5	40.1															23.4	36.0			
			WINTER		5.7						3.7		10.1								80.5			
Hg4470	M	2012	SPRING					7.3			18.9			8.2			8.5		21.5	20.0	15.5			
			WINTER					23.4		46.2											30.4			
Hg4675	M	2012	SPRING		2.1		0.7	0.8			55.3				7.3		3.0		0.7	19.4	10.7			
			WINTER		2.2					4.2	54.8		5.4	15.0			7.1		6.1		5.3			
Hg4792	M	2012	SPRING	1.4							26.7		17.5		3.6						50.9			
			WINTER						0.8	7.0											74.5	17.7		
Hg5692	M	2012	SPRING	0.4		9.1							41.1								49.5			
			WINTER							17.7											72.4	9.9		
Hg6231	M	2012	SPRING	1.7		10.5		6.8			44.3		24.4								12.4			
			WINTER					3.2	1.1	28.0	58.7										9.0			
Hg11043	M	2013	SPRING	1.1	59.9															20.4	18.6			
			WINTER		15.4							8.1	52.7								23.7			
Hg11044	M	2013	SPRING	5.6		76.1					8.6									9.7				
			WINTER												3.0						91.9	5.1		
Hg11045	M	2013	SPRING	4.0	42.0	0.5															53.6			
			WINTER		3.3			9.5			2.5		18.3								66.5			
Hg11049	M	2013	SPRING	1.8		66.8					8.0									17.7	5.7			
			WINTER		5.7		0.9			18.3	56.6							3.9	5.8	6.7	0.1			2.0
Hg4237	M	2014	SPRING	1.1																	98.9			
			WINTER		1.2			16.9													81.9			
Hg4819	M	2014	SPRING	0.6																	99.4			
			WINTER												2.2						97.8			
Hg5156	M	2014	SPRING	2.9		6.7		9.4			17.1										63.9			
			WINTER						1.9	2.0					4.6						89.5	2.0		
Hg5605	M	2014	SPRING	3.5		32.8															63.7			
			WINTER					23.0			14.2			17.8							45.0			
Hg12	F	1993	SPRING	0.2	12.6	8.7							47.4			4.1					27.0			
			WINTER		5.6			3.3		65.1	11.5										14.5			
Hg184	F	1993	SPRING							60.2							2.5				37.3			
			WINTER		0.4			0.0	9.7	58.0							4.6				27.3			
Hg3600	F	1993	SPRING	3.3		37.6															59.1			
			WINTER								46.1				0.2						53.7			
Hg89	F	1994	SPRING			1.8		7.7		32.0	18.7		1.7		1.8		0.9				35.3			
			WINTER								18.5		9.7								67.6			4.3
Hg583	F	1994	SPRING	2.0		6.2		2.0					42.9	14.9							32.0			
			WINTER		4.0		3.4	9.4			20.6		15.6				6.8		14.7	2.0	20.6		2.9	
Hg3605	F	1994	SPRING					43.6		11.6			15.0	2.9	3.3						23.6			
			WINTER					9.8	11.4	1.6	44.7			10.9			12.8				8.8			
Hg3607	F	1994	SPRING	0.5		9.7		22.1			7.3		15.1		4.9		14.3				26.1			
			WINTER					9.4		47.5	11.0										32.1			
Hg21	F	1995	SPRING	0.9	12.6			40.9		2.6					11.8						31.2			
			WINTER		7.0			43.4							0.8						48.8			
Hg2425	F	1995	SPRING		0.1			49.6		37.8											12.4			
			WINTER		1.1			18.9		15.1											64.9			
Hg2575	F	1995	SPRING	0.1	4.6	15.1							17.8		0.0	17.8	7.2			18.0	19.5			
			WINTER		0.8				25.3	54.2											18.4	1.2		
Hg2595	F	1995	SPRING		32.1			20.1							2.0						45.8			

			WINTER	1.2	2.9			63.5													32.4		
Hg2604	F	1995	SPRING					25.3		28.1					6.2						40.4		
			WINTER		9.1			18.1		16.6					11.9						44.4		
Hg23	F	1996	SPRING		4.9			24.5		48.5											22.1		
			WINTER		1.0			31.5		50.9											16.6		
Hg2985	F	1996	SPRING		14.4					36.3					1.6						47.7		
			WINTER		17.1						40.7		38.4								3.9		
Hg2986	F	1996	SPRING	3.4		32.5							23.6		1.8						38.8		
			WINTER		0.2			0.4		64.3											35.1		
Hg3021	F	1996	SPRING					11.8		19.8					8.2						60.2		
			WINTER		5.0				31.5		16.9			25.0			13.3				8.4		
Hg3055	F	1996	SPRING		4.3			8.8		34.6											52.3		
			WINTER						3.3	46.4	4.8						11.5				34.0		
Hg3071	F	1996	SPRING		25.3					21.4					2.8						50.5		
			WINTER		4.6			45.1		6.0											33.7	10.6	
Hg3076	F	1996	SPRING		10.5			11.5		46.0											32.1		
			WINTER					27.7		37.8	10.9										23.6		
Hg26	F	1997	SPRING	2.1				1.6			31.4			20.3						26.8	17.9		
			WINTER		8.9			1.4			15.5			26.0							3.8	8.6	35.8
Hg757	F	1997	SPRING	1.3		36.2								7.3		27.3					27.9		
			WINTER		7.3				25.3	11.5	19.5										36.5		
Hg3612	F	1997	SPRING	1.9		17.9		19.2		25.4	4.9										30.7		
			WINTER		8.0			13.3		37.6											41.1		
Hg3618	F	1997	SPRING	6.1		42.8															51.1		
			WINTER		5.6				12.5	37.1											44.8		
Hg3619	F	1997	SPRING		0.4			15.4		48.8											35.4		
			WINTER		36.9			57.9			5.2												
Hg59	F	1998	SPRING		2.1				1.1	69.1											27.8		
			WINTER							77.6											22.4		
Hg36	F	1998	SPRING		4.6			13.7	0.8	52.9					1.2						26.8		
			WINTER		8.2			15.8	9.8	42.5	8.1						5.1				10.5		
Hg2691	F	1999	SPRING					6.5		23.9	9.7		21.5	0.0	0.3						38.0		
			WINTER						3.1	44.4	6.2						11.3				35.0		
Hg2747	F	1999	SPRING		22.5			14.8		30.3											32.5		
			WINTER					40.8		44.0											15.2		
Hg5098	F	1999	SPRING		19.3			16.7						17.8	6.4						39.9		
			WINTER					3.8		39.2	17.7		21.2	0.5							17.6		
Hg7364	F	2004	SPRING		6.2				1.4	42.5											49.8		
			WINTER					23.1		10.2	17.3		11.6		2.1						35.8		
Hg7366	F	2004	SPRING		4.4			7.0		43.7											45.0		
			WINTER							3.5	4.9		32.0		2.4						57.2		
Hg7384	F	2004	SPRING		1.6			15.3		39.1											44.0		
			WINTER		0.2			31.8			30.5		0.1	0.2	6.6						30.7		
Hg137	F	2011	SPRING		10.1			23.2													66.7		
			WINTER		1.9			47.0													51.1		
Hg2718	F	2011	SPRING		1.2			42.8							6.2						49.8		
			WINTER		0.9			45.1							2.5						51.4		
Hg2999	F	2011	SPRING			27.7		22.0													50.3		
			WINTER		14.6			60.4													25.0		
Hg3736	F	2011	SPRING		17.2				3.1	20.8											58.9		
			WINTER		4.0			39.8		18.8											37.5		
Hg5846	F	2011	SPRING		9.9	10.2		33.5													46.4		
			WINTER		33.5			66.5															
Hg6630	F	2011	SPRING		6.0					31.0					1.1						61.9		
			WINTER		2.1			11.0		35.2											51.6		
Hg10322	F	2011	SPRING		3.6			24.6													71.9		
			WINTER					23.7		9.2	11.6		8.1	3.0							44.4		
Hg10324	F	2011	SPRING	1.5		30.3							36.4								31.9		
			WINTER		0.2						6.2		23.5								70.2		
Hg10325	F	2011	SPRING		8.4	3.7		29.4													58.5		
			WINTER		7.2			59.7													33.2		
Hg10327	F	2011	SPRING		14.4			0.7		19.7					2.6						62.7		
			WINTER		2.5			9.1		35.0											53.4		
Hg10328	F	2011	SPRING	0.8				27.6			7.0		13.4								51.3		
			WINTER		2.8			23.1			29.1				4.9						40.0		
Hg10330	F	2011	SPRING		6.5	8.6		49.6													35.3		
			WINTER		10.2			64.2													25.6		
Hg10331	F	2011	SPRING		6.6			47.8													45.5		
			WINTER		12.1			75.0													12.9		
Hg10333	F	2011	SPRING		14.6			38.1						5.5	3.4						38.4		

			WINTER		2.9		56.5					16.9		3.3		20.4	
Hg10334	F	2011	SPRING		12.7		27.8									59.5	
			WINTER		6.6		40.8									52.6	
Hg102	F	2012	SPRING		9.4		60.3		2.8							27.5	
			WINTER		0.4		42.3		14.8	28.2						14.3	
Hg109	F	2012	SPRING		6.4		53.2		16.3							24.1	
			WINTER	0.1	2.8		12.9			51.0	15.6					17.6	
Hg3616	F	2012	SPRING		17.2		39.1		5.0							38.7	
			WINTER		5.5		5.4		15.8	12.6						60.8	
Hg4266	F	2012	SPRING		13.2		48.0									38.9	
			WINTER				44.4		15.4	20.0						15.4	4.8
Hg9019	F	2012	SPRING		14.7		47.8									37.6	
			WINTER				52.6		4.2	25.2						18.0	
Hg10687	F	2012	SPRING				37.5		19.1			20.8				22.7	
			WINTER		2.2		36.8		17.8	27.1	7.7					8.5	
Hg10688	F	2012	SPRING				42.5		21.8							35.8	
			WINTER		4.0		9.8		2.5	41.0	21.7					21.0	
Hg10689	F	2012	SPRING				32.4		19.3							48.2	
			WINTER		2.5		30.9									66.7	
Hg10690	F	2012	SPRING		16.7		43.4		5.1							34.8	
			WINTER		2.6		60.2		21.4							15.8	
Hg45	F	2013	SPRING		13.5		32.1									54.5	
			WINTER		15.2		9.9			10.5	6.9					57.5	
Hg3074	F	2013	SPRING		30.1		0.8	0.7	6.0				0.7			61.6	
			WINTER		0.5		2.2	3.1	18.6							75.7	
Hg5485	F	2013	SPRING		22.0	15.9	8.6									53.5	
			WINTER		37.2	18.6	20.2			5.4					3.1	15.5	
Hg11042	F	2013	SPRING		14.9		18.2									66.9	
			WINTER		5.3		41.4				16.2					37.2	
Hg11051	F	2013	SPRING		25.8		8.1	1.8	2.2							62.0	
			WINTER		4.8		42.8		13.0							39.5	
Hg69	F	2014	SPRING				16.4		30.0							53.7	
			WINTER				12.2		19.4	25.3						43.1	
Hg6177	F	2014	SPRING		11.7		31.1									57.2	
			WINTER				43.1		1.1							55.8	
Hg8389	F	2014	SPRING	0.7	12.4		66.1						1.4			19.4	
			WINTER		1.4		68.8		17.3							12.5	
Hg11977	F	2014	SPRING		0.4		14.8		41.4							43.5	
			WINTER				13.8		42.1							44.1	
Hg11978	F	2014	SPRING				57.5									42.5	
			WINTER				67.3									32.7	
