# Supplementary material for: Measuring repeatability of compositional diet estimates: An example using quantitative fatty acid signature analysis
Source: Ecol Evol. 2022 Oct 27;12(10):e9428. doi: 10.1002/ece3.9428 (PMC9608821; doi:10.1002/ece3.9428)
Supplement: Supplementary file 2 — Table S2 [file ECE3-12-e9428-s002.pdf]

Table A2. QFASA diet estimates for 78 adult grey seals (36 males, 42 females) sampled in September-October (FALL) and then again during the subsequent breeding season (December-January, WINTER). M, male; F, female.

Seal ID	Sex	Year	Season	Atlantic Butterfish	Capelin	Atlantic Herring	Atlantic Mackerel	Northern Sandlance	Snake Blenny	Atlantic Cod	Pollock	Silver Hake	White Hake	American Plaice	Winter Flounder	Witch Flounder	Yellowtail Flounder	Smooth Skate	Thorny Skate	Winter Skate	Redfish	Longhorn Sculpin	Sea Raven	Northern Shortfin Squid
Hg2667	M	1995	FALL	0.3	5.7		1.5	12.2			36.2		30.2						4.5	3.8	5.7			
			WINTER		5.6			7.3		25.4	45.8				0.1				4.1	5.7	6.1			
Hg3096	M	1996	FALL		3.2								63.4		3.7						29.7			
			WINTER		0.0					22.1			47.7		7.8						22.5			
Hg3099	M	1996	FALL					17.9		18.4					9.0		0.0				54.7			
			WINTER					14.2		37.0					3.2						45.7			
Hg3100	M	1996	FALL								40.5		45.0								14.5			
			WINTER		3.0			9.6		28.6	30.4		21.5								6.9			
Hg3101	M	1996	FALL		7.1					4.4	26.6		52.0								9.9			
			WINTER		4.3					16.7	29.7		31.1								18.3			
Hg3104	M	1996	FALL		4.1			10.9			40.4		33.3				5.8			2.8	2.7			
			WINTER		6.0			12.4		30.0	38.8		4.0							6.0	2.8			
Hg623	M	1997	FALL							21.9			0.5				35.7				41.9			
			WINTER		1.6			0.6		4.3	3.5		59.8	0.0	1.6						28.8			
Hg3080	M	1997	FALL							7.9	29.1			21.0	5.3		5.0				31.6			
			WINTER					1.7	10.7	32.0	25.1			2.5							28.0			
Hg3737	M	1997	FALL		2.9			4.4	11.4	54.0											27.3			
			WINTER					1.8	0.7	54.6	13.1										29.8			
Hg5111	M	1999	FALL					37.7		19.5				18.7	2.3						21.9			
			WINTER		6.2			27.4		28.9	18.4		2.2	0.2	3.5						13.2			
Hg5112	M	1999	FALL	1.0	17.7								47.7								33.7			
			WINTER					6.9		37.7	7.1										48.3			
Hg5114	M	1999	FALL		1.4						23.6		60.5		1.7	0.0	5.7				7.2			
			WINTER		5.6					35.1	43.3				8.8					2.5	4.8			
Hg5116	M	1999	FALL		3.6			19.4		16.1	18.1		35.8								7.1			
			WINTER		2.1			12.1		29.3	20.5		31.0	0.2							4.7			
Hg2777	M	2000	FALL						4.5		47.7		23.0				13.0				11.8			
			WINTER		0.5				2.3		43.3		20.4	13.2							9.7	4.7	6.0	
Hg5684	M	2000	FALL	2.0	3.1			3.7			51.7		20.4		13.5				0.6		5.1			
			WINTER		4.6					3.8	59.1				9.4				4.3	13.9	5.0			
Hg5685	M	2000	FALL		2.6			14.8		29.0	29.5		4.0								20.1			
			WINTER		2.1			18.3		26.2	35.1										18.3			
Hg5686	M	2000	FALL		9.1			18.7			4.8		53.8								13.6			
			WINTER		2.8			7.4		21.2	17.7		40.8								10.2			
Hg5687	M	2000	FALL		2.6			9.7		5.9	26.5		47.0								8.3			
			WINTER		0.2			24.9		20.0	36.0		14.7								4.1			
Hg3661	M	2000	FALL	0.8	9.2						30.7		0.0			13.5	0.2			41.8	3.9			
			WINTER		11.5			5.7		17.4	34.7				1.4					25.9	1.6		1.9	
Hg6124	M	2001	FALL	0.3				13.1		12.0	16.6		38.7	0.8	3.0						15.6			
			WINTER					1.2		0.5	40.3			11.3	13.0						33.8			
Hg6125	M	2001	FALL					16.1			17.2			24.2			19.8				22.8			
			WINTER					2.6			42.2			25.5	7.5		3.5				18.6			
Hg6126	M	2001	FALL		10.2		1.1	6.5			64.1				0.8				11.4	6.0	0.0			
			WINTER		6.5			23.1			48.2				3.1					19.1				
Hg6123	M	2001	FALL								7.5		11.3	28.4							52.8			
			WINTER					13.9		6.2	9.5			19.7	1.9						48.8			
Hg2792	M	2009	FALL		1.8			9.5		14.5	22.6		38.2				2.8		1.4		9.3			
			WINTER		5.3			2.4			55.9				1.5				1.6	24.3	9.1			
Hg4057	M	2009	FALL	2.5	3.2			27.6			30.0		20.7	6.5							9.4			
			WINTER		1.5			29.2		6.9	23.6			26.8	2.2						9.9			
Hg6900	M	2009	FALL		6.9						21.6	14.0	43.0								14.4			
			WINTER		13.2					3.5	38.1		29.3								10.0			6.0
Hg9022	M	2009	FALL		11.5						15.7	2.7	46.3								16.1			7.6
			WINTER		9.3						17.2	0.8	54.1								13.2			5.5
Hg9413	M	2009	FALL		9.2						13.7	18.1	34.3								21.6			3.1
			WINTER		2.1						25.5	1.6	45.4								23.4			1.9
Hg9415	M	2009	FALL		11.0					17.4	39.3		12.7								19.6			
			WINTER		4.6			5.7		24.7	27.0		12.9								25.2			
Hg9416	M	2009	FALL		3.3			57.1		1.0	9.5		21.0								8.0			
			WINTER		1.6			49.1		6.3	17.3		14.0								11.7			
Hg3648	M	2010	FALL	1.9	5.6			1.6			51.2		6.6								33.2			
			WINTER	1.2	1.3			2.7			27.7		48.2								18.9			
Hg4515	M	2010	FALL					10.8		19.7	6.5				0.6						62.5			
			WINTER					4.2		16.5	24.0										55.3			
Hg5954	M	2010	FALL								6.1				5.4						88.5			
			WINTER							23.4	9.3										67.3			
Hg6195	M	2010	FALL	0.8	9.1	2.5					49.8		22.7							0.2	14.9			
			WINTER		10.8	3.9		47.3			8.2		19.5								10.3			
Hg9935	M	2010	FALL	0.5	4.1					3.3	46.4		24.4		1.5						19.9			
			WINTER		14.6						15.3		58.9								11.2			
Hg9936	M	2010	FALL		6.4			3.8	24.8	3.7	21.2										17.0	19.0	4.1	
			WINTER		6.2			20.2	4.0		29.8						4.8				11.0	24.0		
Hg19	F	1995	FALL		2.4			49.0		22.5											23.0	3.1		
			WINTER					42.6		19.0	9.7			13.7							15.0			
Hg24	F	1995	FALL		12.1			22.4		13.0											49.9	2.7		
			WINTER		1.9			34.8		19.2											44.2			
Hg2668	F	1995	FALL		29.4			13.2													50.6	6.8		
			WINTER		4.3			60.6													35.1			
Hg574	F	1996	FALL					8.1		38.7											53.2			

			WINTER				20.5		28.0	0.4					51.1		
Hg2689	F	1996	FALL				23.5		11.0				4.2		61.3		
			WINTER				31.0		18.4						50.6		
Hg3095	F	1996	FALL		8.8		21.0		32.3						35.4	2.6	
			WINTER				34.2		35.2	2.7					27.9		
Hg3098	F	1996	FALL		19.3		35.6		27.6							17.4	
			WINTER		7.9		33.9		49.7							8.5	
Hg3103	F	1996	FALL		15.1		39.8		14.9							30.2	
			WINTER				32.7		9.1	24.0		19.5		9.3	5.4		
Hg146	F	1997	FALL		19.6		9.8		61.0						9.0	0.6	
			WINTER		10.1		25.0		59.0						5.8		
Hg3735	F	1997	FALL		0.8			14.8	47.8						36.5		
			WINTER				1.1		50.2						48.8		
Hg125	F	1999	FALL				44.4		25.5			5.2			25.0		
			WINTER				40.7		43.3						16.1		
Hg3804	F	1999	FALL		7.0		32.5								50.2	10.3	
			WINTER				33.6		26.0						40.4		
Hg5108	F	1999	FALL		18.3		12.0								61.1	8.6	
			WINTER				31.3		10.6				2.7		55.5		
Hg5110	F	1999	FALL		7.4		37.0		12.2						35.1	8.3	
			WINTER		6.1		21.2		7.6		45.8				19.3		
Hg2573	F	2000	FALL		3.6				38.1						54.8	3.6	
			WINTER						13.1	28.8	9.1				49.1		
Hg3271	F	2000	FALL		12.6		36.2		28.7						22.5		
			WINTER		4.4		80.5		8.7						6.5		
Hg5681	F	2000	FALL				44.2		6.7				0.6		47.4	1.1	
			WINTER				64.1		16.4						19.5		
Hg5683	F	2000	FALL		2.9		15.0		48.4						33.7		
			WINTER				23.1		45.1						27.0		4.8
Hg2690	F	2001	FALL		8.5		41.4		18.0						27.1	5.1	
			WINTER				60.2		4.0						31.4	4.4	
Hg6116	F	2001	FALL		10.3		45.7		18.1						12.8	13.2	
			WINTER				39.6			35.8		1.6		16.2	6.8		
Hg6118	F	2001	FALL		17.7										70.0	12.3	
			WINTER		3.9		26.3			14.5					55.4		
Hg6120	F	2001	FALL		18.1		17.7		10.9						45.5	7.8	
			WINTER		6.9		41.9		5.5						45.6		
Hg6122	F	2001	FALL		20.0		10.8								61.6	7.7	
			WINTER		6.2		39.2								49.4	5.2	
Hg93	F	2009	FALL		11.0		52.4								36.6		
			WINTER		1.6		57.1				0.0				41.3		
Hg829	F	2009	FALL		21.9		52.2								25.9		
			WINTER				61.1			4.0					34.9		
Hg2734	F	2009	FALL		14.8		66.4								18.9		
			WINTER		15.7		50.2			21.5					12.6		
Hg4269	F	2009	FALL		15.1		41.0								43.3	0.6	
			WINTER		7.7		54.5								35.4	2.3	
Hg6165	F	2009	FALL		15.5		30.7								53.9		
			WINTER		13.9		11.0			33.0	14.0				28.1		
Hg9410	F	2009	FALL		9.1		46.3								44.6		
			WINTER		4.1		40.2			22.1					33.7		
Hg9417	F	2009	FALL		7.2	0.4	31.8		17.3						43.3		
			WINTER	0.6	18.3	2.5				25.1	28.6				24.9		
Hg9418	F	2009	FALL		6.8		16.0						1.5		75.7		
			WINTER		58.9					11.4	29.7						
Hg9420	F	2009	FALL		16.0		42.3								41.7		
			WINTER		11.5		36.6			16.6	7.1				28.2		
Hg78	F	2010	FALL				23.0			0.5	13.2		1.1	8.4	53.7		
			WINTER	1.9	8.3					18.3	23.2				48.3		
Hg9928	F	2010	FALL		6.6		51.3								42.1		
			WINTER	2.3	13.4					51.0	0.7				32.6		
Hg9930	F	2010	FALL		15.5		17.0						1.5		66.0		
			WINTER		16.3					19.7	19.2				44.9		
Hg9931	F	2010	FALL	0.5			20.8		18.4						60.3		
			WINTER	0.1			9.1						5.8		85.1		
Hg9932	F	2010	FALL		23.6		6.0	1.2	20.2						49.1		
			WINTER		3.6		20.8		23.4						52.2		
Hg9933	F	2010	FALL		1.8			6.0	4.1						88.0		
			WINTER		16.7						10.9				72.4		
Hg9934	F	2010	FALL		11.9		12.1	2.4							73.6		
			WINTER				16.3		7.3	16.3					60.2		
Hg9939	F	2010	FALL	2.2	1.6					26.5	9.0				60.6		
			WINTER				31.0	0.5	7.8				2.0		58.7		
Hg30	F	2012	FALL		16.1		45.3		0.5						38.0		
			WINTER		0.2		58.4		11.2	8.7					21.5		
Hg142	F	2012	FALL		12.1		2.2		13.8						71.9		
			WINTER				27.7		17.5						54.8		
