# Supplementary material for: Measuring repeatability of compositional diet estimates: An example using quantitative fatty acid signature analysis
Source: Ecol Evol. 2022 Oct 27;12(10):e9428. doi: 10.1002/ece3.9428 (PMC9608821; doi:10.1002/ece3.9428)
Supplement: Supplementary file 3 — Table S3 [file ECE3-12-e9428-s003.pdf]

Table A3. QFASA diet estimates for 24 adult grey seal females sampled during the breeding season (December-January, WINTER) in multiple years (2-5) between 1999 and 2006.

Seal ID	Year	Atlantic Butterfish	Capelin	Atlantic Herring	Atlantic Mackerel	Northern Sandlance	Snake Blenny	Atlantic Cod	Pollock	Silver Hake	White Hake	American Plaice	Winter Flounder	Witch Flounder	Yellowtail Flounder	Smooth Skate	Thorny Skate	Winter Skate	Redfish	Longhorn Sculpin	Sea Raven	Northern Shortfin Squid
Hg12	1999					19.3		43.6											37.1			
	2002							35.6	17.8		4.4								42.3			
Hg32	2000					1.3		51.8	10.8										36.1			
	2002					2.1		44.9											53.0			
Hg3263	1999		5.1			13.2		55.4											25.5			0.9
	2001							59.8											40.2			
Hg3250	1999						5.4	27.6	43.3				1.5						22.2			
	2000		8.5			51.1		7.8				14.0							18.5			
	2002					6.3		48.0											45.7			
Hg6035	2001		6.3			45.2		7.5	8.0			24.4							8.6			
	2002		4.5	4.5	0.4	30.0			11.5			32.1					4.5		12.4			
Hg4388	1999		1.8						39.7		12.3								46.1			
	2000					6.5		45.2											47.0			1.3
	2002							36.0											61.1			2.9
Hg3994	1999								36.4		7.5								56.2			
	2000								4.7		32.3								63.0			
	2001								2.4		28.0								69.7			
Hg4374	1999	1.5	17.9			35.6			45.0													
	2001		9.2								14.7								76.1			
	2003		19.1								1.8								79.1			
Hg3817	1999					31.2	1.5	14.6	41.6										11.1			
	2000		0.7			29.7			34.5		10.7								24.4			
	2001		0.4			46.4	0.7		47.9										4.7			
	2002					53.0		24.9											20.3	1.8		
Hg505	1999							63.4	11.4										15.2	10.0		
	2000							45.0	15.4			0.8			4.5				22.6	11.7		
	2002							22.1			45.9		0.5						31.5			
	2003										43.8								53.4			2.8
Hg4735	1999		9.2			54.0			24.1						6.8				5.9			
	2001		4.8			25.6		30.7											38.8			
	2002		2.2			37.6		19.8											40.4			
	2003					22.7			19.4										53.0			5.0
Hg132	1999					32.3		48.5	6.0										13.2			
	2001					16.0		30.7	25.3		0.7								27.2			
	2002					6.3		31.3											62.4			
	2004					5.3		23.9	10.4										60.4			
Hg4393	1999		2.0			2.1	0.0	2.1	44.2						12.0				37.6			
	2000						1.0		41.7		11.6		0.5		9.3				36.0			
	2001										30.0		1.6					9.2	59.2			
	2002		16.9						20.5		45.0								17.6			
Hg4489	1999						19.6				32.9								46.8			0.7
	2001						2.3	14.7			24.7		5.5						52.9			
	2002							20.9			13.5		1.6						64.0			
	2003										25.4		8.6						66.0			
	2006						7.5	2.4			14.9	11.5	15.8						47.9			
Hg4491	1999		8.6						24.3		20.6								46.6			
	2000								2.9		15.9				9.1				72.2			
	2001										18.7		0.6						80.7			
	2002												3.6					9.8	86.6			
	2003		10.1																87.1			2.9
